# 5-HT2B Receptor Antagonists Inhibit Fibrosis and Protect from RV Heart Failure

**DOI:** 10.1155/2015/438403

**Published:** 2015-02-01

**Authors:** Wiebke Janssen, Yves Schymura, Tatyana Novoyatleva, Baktybek Kojonazarov, Mario Boehm, Astrid Wietelmann, Himal Luitel, Kirsten Murmann, Damian Richard Krompiec, Aleksandra Tretyn, Soni Savai Pullamsetti, Norbert Weissmann, Werner Seeger, Hossein Ardeschir Ghofrani, Ralph Theo Schermuly

**Affiliations:** ^1^Universities of Giessen and Marburg Lung Centre (UGMLC), Aulweg 130, 35392 Giessen, Germany; ^2^German Center for Lung Research (DZL), 35392 Giessen, Germany; ^3^Max-Planck-Institute for Heart and Lung Research, Ludwigstrasse 43, 61231 Bad Nauheim, Germany

## Abstract

*Objective*. The serotonin (5-HT) pathway was shown to play a role in pulmonary hypertension (PH), but its functions in right ventricular failure (RVF) remain poorly understood. The aim of the current study was to investigate the effects of Terguride (5-HT2A and 2B receptor antagonist) or SB204741 (5-HT2B receptor antagonist) on right heart function and structure upon pulmonary artery banding (PAB) in mice. *Methods*. Seven days after PAB, mice were treated for 14 days with Terguride (0.2 mg/kg bid) or SB204741 (5 mg/kg day). Right heart function and remodeling were assessed by right heart catheterization, magnetic resonance imaging (MRI), and histomorphometric methods. Total secreted collagen content was determined in mouse cardiac fibroblasts isolated from RV tissues. *Results*. Chronic treatment with Terguride or SB204741 reduced right ventricular fibrosis and showed improved heart function in mice after PAB. Moreover, 5-HT2B receptor antagonists diminished TGF-beta1 induced collagen synthesis of RV cardiac fibroblasts *in vitro*. *Conclusion*. 5-HT2B receptor antagonists reduce collagen deposition, thereby inhibiting right ventricular fibrosis. Chronic treatment prevented the development and progression of pressure overload-induced RVF in mice. Thus, 5-HT2B receptor antagonists represent a valuable novel therapeutic approach for RVF.

## 1. Introduction

Sustained pressure overload of the right ventricle (RV) is a significant pathophysiological factor in several cardiovascular disorders, including pulmonary hypertension (PH). Outstandingly, RV failure (RVF) is the most common cause of death in patients with severe PH and is increasingly recognized as an important clinical problem [1, 2].

Long-term increase in pressure consequently results in RV hypertrophy (RVH). Initial cardiac hypertrophy can be considered as a beneficial response to changed hemodynamic parameters. During disease progression the maladaptive cardiac hypertrophy slowly proceeds to a decompensated state. With the progression of PH, the RV dilates, becomes fibrotic, and finally undergoes functional failure, the ultimate cause of death in PH [3].

An accumulating body of evidence clearly underlines a significant role of 5-hydroxytryptamine (serotonin, 5-HT) in development and progression of LV hypertrophy (LVH) and fibrosis [4–6]. It was shown that serotonin, via the 5-HT2B receptor (5-HT2BR), regulates cardiac development and function [7, 8]. Also it was demonstrated that 5-HT2BR is essential for isoproterenol-induced cardiac hypertrophy, which is regulated by interleukin-6 (IL-6), interleukin-1*β* (IL-1*β*), and tumor necrosis factor *α* (TNF*α*) cytokine production by cardiac fibroblasts [9]. Moreover, 5-HT2BR blockade has also been shown to prevent cardiac hypertrophy induced by angiotensin II or isoproterenol infusion [10]. In 2009, Jaffré et al. found that the expression of 5-HT2BR by noncardiomyocytes is required for isoproterenol-induced cardiac hypertrophy and that 5-HT2BR is overexpressed in hearts of patients with congestive heart failure [11]. In addition to that, several research groups revealed a role for serotonin in the pathogenesis of PAH [12–16]. Regarding different forms of chronic LV failure (LVF) extensive experimental knowledge was achieved over the past decades. Although the concepts of mechanisms of RVF have been largely formed on the basis of the analysis of LVF models, thus the molecular processes contributing to the maintenance and disease of the RV are poorly investigated. We therefore investigated the effects of the selective 5-HT2BR antagonist (SB204741) and a combined 5-HT2AR and 5-HT2BR antagonist (Terguride) on RVH and fibrosis in a preclinical animal model of RV pressure overload. First, we examined the expression of the 5-HT2AR and 5-HT2BR in the mouse model of RV pressure overload, induced by pulmonary artery banding (PAB). Importantly, only 5-HT2BR expression was elevated after PAB. Our data show that the 5-HT2BR antagonist SB204741 and antagonist of both 5-HT2A and 2B receptors Terguride prevent RV myocardial hypertrophy. Furthermore, both antagonists exerted antifibrotic effects after long-term use in chronic experimental pressure overload-induced RVF in mice. Analyses of RV cardiac fibroblasts treated with 5-HT2BR antagonists substantiated the role of the 5-HT2BR in collagen production.

## 2. Methods

### 2.1. Animals

Adult male C57BL/6J mice (body weight 20–23 g) were purchased from Charles River Laboratories (Sulzfeld, Germany). Animals were housed under controlled conditions with free access to rodent chow and tap water. All* in vivo* procedures were approved by the Animal Ethics Committee of the Regierungspräsidium Darmstadt (Az. B2/229) in accordance with institutional guidelines that comply with national and international regulations.

### 2.2. Animal Experimental Design

The effects of 5-HT signaling pathway blockade by 5-HT2B receptor (5-HT2BR) antagonists were investigated in mice after banding of the main pulmonary artery (PAB). For the experiment, mice were randomly assigned for placebo or chronic Terguride or SB204741 therapy: (1) sham-operated animals, (2) mice subjected to PAB with no active treatment, designated as placebo group, (3) mice subjected to PAB with Terguride administration (Terguride group), and (4) mice subjected to PAB with SB204741 administration (SB204741 group). Administration of both compounds was performed from 7 to 21 days after surgery. Doses of Terguride and SB204741 were chosen according to preceding pilot experiments, addressing specificity and tolerability of these agents (0.2 mg/kg/d for Terguride and 5 mg/kg/d for SB204741).

### 2.3. PAB-Induced RHF and Treatment with 5-HT2BR Antagonists

Adult male C57BL/6J mice (Charles River Laboratories, Sulzfeld, Germany) were subjected to banding of the main pulmonary artery (PAB) or sham operation under isoflurane anesthesia (1.5% vol/vol) and a subcutaneous administration of 0.03 mg/kg buprenorphine hydrochloride. The mice were intubated and respiration was controlled by a rodent ventilator (Harvard Apparatus, USA). The left thorax was opened at the third intercostal space to expose the pulmonary artery. The pulmonary artery was carefully dissected free from the ascending aorta and a surgical hemoclip was positioned around the pulmonary artery leaving the vessel constricted to a diameter of 0.35 mm. The thorax was then closed with Vicryl suture. Sham-operated animals underwent the same surgical procedure except for the artery constriction. Long-term treatment was administered by intraperitoneal injection. Terguride was dissolved in ethanol and subsequently diluted with hydrochloric acid prior to pH adjustment to 7.4. SB204741 was dissolved in ethanol and subsequently diluted with hydrochloric acid prior to pH adjustment to 7.4. Placebo groups received ethanol/saline solution at the same volume.

### 2.4. Hemodynamic Assessment

Twenty-one days after pulmonary artery banding, the mice were anaesthetized by inhalation of isoflurane (1.5% vol/vol). Core body temperature was maintained at 37°C using a controlled heating pad. A Millar microtip catheter (SPR-671, FMI, Foehr Medical Instruments GmbH, Seeheim/Ober-Beerbach, Germany) was inserted through the right jugular vein into the right ventricle for measurement of RV pressure. Afterwards the same catheter was inserted into the left carotid artery to measure systemic arterial pressure. All hemodynamic measurements were performed with a PowerLab system using the LabChart 7.0 software (ADInstruments GmbH, Spechbach, Germany). The following parameters were calculated: RV systolic pressure (RVP_sys_), systolic and diastolic systemic blood pressure (SBP_sys_, SBP_dias_), and heart rate (HR).

Following the hemodynamic measurements, mice were killed by exsanguination; right ventricles (RVs) were separated from left ventricles and septum (LV + S). The organs were weighed and the tibia length was measured. RVs and LV + Ss were either quickly frozen in liquid nitrogen for RNA extraction or fixed in 3.5% to 3.7% formalin for histological quantification of collagen content.

### 2.5. Magnetic Resonance Imaging

For analysis of 5-HT2BR blockade, sham-operated (*n* = 9), PAB-operated (*n* = 9), and Terguride (*n* = 6) and SB204741 (*n* = 6) treated animals underwent MRI investigation at day 21 after surgery. For cardiac MRI measurements a 7.0 T Bruker PharmaScan, equipped with a 300 mT/m gradient system, a custom-built circularly polarized birdcage resonator, and the IntraGate self-gating tool (Bruker, Ettlingen, Germany), was used. Measurements were done under isoflurane anesthesia (2.0% vol/vol) and body core temperature was maintained at 37°C. For gradient echo technique the following parameters were adjusted: repetition time = 6.2 ms; echo time = 1.6 ms; field of view = 2.20 × 2.20 cm; slice thickness = 1.0 mm; matrix = 128 × 128; repetitions = 100; resolution = 0.0172 cm/pixel. The imaging plane was localized using scout images showing the sagittal and coronal view of the heart, followed by acquisition in axial view, orthogonally to the septum of both scout scans. Multiple contiguous axial slices were acquired for complete coverage of the left and right ventricle. MRI data was analyzed using MASS 4Mice digital imaging software (Medis, Leiden, Netherlands).

### 2.6. Gene Expression by RT-qPCR

RV homogenates were subjected to gene expression analysis of 5-HT2AR and 5-HT2BR. For this purpose, total RNA extraction, cDNA synthesis, and quantitative (q) RT-PCR were performed. Primers were designed using the online Invitrogen primer design tool. According to known mouse sequences, the primers for 5-HT2AR (5′-CCAGAACCAAAGCCTTCCTG-3′ and 5′-CCATGATGGTTAGGGGGATG-3′) and 5-HT2BR (5′-CAGGCCAAT-CAGTGCAACTC-3′ and 5′-AAGCGGTCCTTTGTC-AGCTC-3′) were used for specific fragment amplification. Under identical cycling conditions, all primer sets worked with similar efficiencies to obtain simultaneous amplification in the same run, as described before. Hypoxanthine phosphoribosyltransferase (HPRT) was used as a reference gene in all RT-qPCR reactions (5′-GCTGACCTGCTGGATTACAT-3′ and 5′-TTGGGGCTGTACTGCT-TAAC-3′). Relative transcript abundance is expressed as a ΔCt value (ΔCt = Ct^reference^ − Ct^target^).

### 2.7. Determination of Collagen Content in Right Ventricles (RVs)

Freshly dissected RV tissues were fixed in 3.5% to 3.7% formalin overnight, dehydrated, embedded in paraffin, and sectioned (3 *μ*m). To detect collagen fibers, all sections were stained with 0.1% Sirius red F3B (Niepoetter, Bürstadt, Germany) in picric acid (Fluka, Buchs, Germany). Photomicrographs were quantified to determine the interstitial collagen fraction by using Leica QWin V3 computer-assisted image analysis software (Leica Microsystem, Wetzlar, Germany). Average data reflect results from at least five different hearts in each group.

### 2.8. Determination of Collagen Deposition in Isolated Cardiac Fibroblasts

Cardiac fibroblasts (CFs) were isolated from murine right ventricular heart tissues, as described previously [17]. Adult CFs were cultured in DMEM (low glucose medium, 1 g/L), containing 100 U/mL penicillin and 100 *μ*g/mL streptomycin for 24 hours in serum-free conditions before stimulation with TGF-beta 1 (R&D Systems). CFs were treated with 5-HT2BR antagonists SB204741 or Terguride for 1 h prior to TGF-beta 1 (10 ng/mL) stimulation (72 h) at the concentrations indicated. L-Ascorbic acid (0.25 mM) was added to the medium daily.

### 2.9. Statistical Analyses

Values are given as mean ± SEM or as percentages. Parametric data were analyzed by unpaired two-tailed Student's *t*-test or by one-way ANOVA for overall significance, followed by Newman-Keuls' multiple comparison post hoc test. *P* < 0.05 was considered significant.

## 3. Results

### 3.1. Cardiac Expression of 5-HT2AR and 5-HT2BR Receptors in RVF

**Figure 1:**
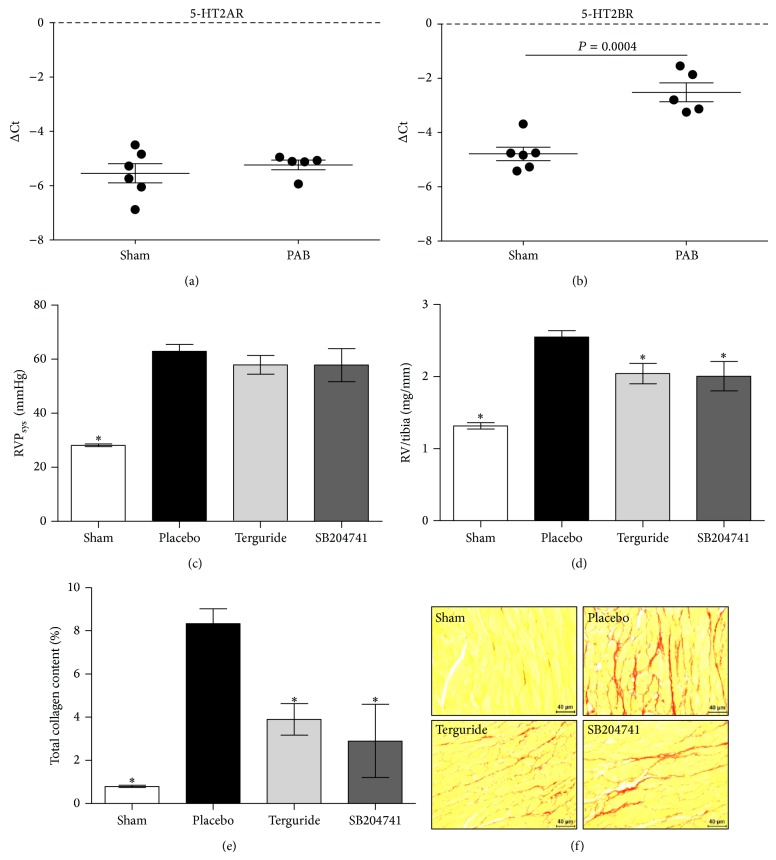
Effect of 5-HT2BR blockade on PAB-induced RVH. mRNA expression of 5-HT2AR (a) and 5-HT2BR (b) in mice right ventricular tissue homogenate 3 weeks after pulmonary artery banding (PAB). While 5-HT2AR expression is unaltered after PAB surgery, 5-HT2BR expression level is significantly increased, in comparison to sham-operated mice. Chronic pulmonary artery constriction led to a sustained and significant increase in RVP_sys_ at day 21 (c). Elevated RV pressure was accompanied by right ventricular hypertrophy measured as ratio of the RV weight to tibia length (d). Chronic treatment from day 7 to day 21 with Terguride (0.2 mg/kg bodyweight) or SB204741 (5 mg/kg bodyweight) reduced right ventricular hypertrophy (c), without having any effect on RVP_sys_ (d). 5-HT2AR and 5-HT2BR blockade decrease PAB-induced collagen deposition as detected by quantitative image analysis of Sirius red staining (e). Representative images are shown in (f). 5-HT2A/B receptor expression and collagen content data represent 5-6 animals for each group. RVP_sys_ and RV/tibia were calculated for *n* = 6–9 animals per group. ^*^ *P* < 0.05 versus placebo-treated control.

To assess whether 5-HT2R is involved in RV failure, we determined the expression levels of both 5-HT2AR and 5-HTBR in RVs 3 weeks after PAB in mice by real-time PCR. Gene expression analysis revealed that both 5-HT2AR and 5-HT2BR were expressed in the RV under basal conditions. In contrast to the 5-HT2AR, whose expression is not significantly changed after the surgery, 5-HT2BR levels were strongly upregulated in RVs from PAB-operated mice compared with sham-operated animals (Figures 1(a) and 1(b)).

### 3.2. Effect of 5-HT2BR Antagonists on Cardiac Performance in Pressure Overload-Induced RVH

Three different experimental set-ups with respective control groups were conducted. Mice subjected to PAB developed progressive RV hypertrophy within 21 days. This is demonstrated by a sustained significant increase in RVP_sys_ to 63.0 ± 10.7 mmHg at day 21 versus 28.1 ± 2.0 mmHg for control animals (*P* < 0.05) (Figure 1(c)). Elevated RV pressure was accompanied by RVH measured as ratio of the RV weight to tibia length. This ratio increased significantly 21 days after surgery to 2.55 ± 0.36 mg/mm in PAB mice versus 1.32 ± 0.17 mg/mm in sham-operated animals (*P* < 0.05) (Figure 1(d)). Daily treatment of PAB-animals from day 7 to day 21 with Terguride or SB204741 attenuated the above-mentioned pathophysiological changes. Treatment of animals with Terguride in a dose 0.2 mg/kg body weight bid did not have any effect on RVP_sys_ (57.9 ± 10.40 mmHg versus 63.0 ± 10.7 mmHg of vehicle treated animals) (Figure 1(c)) but significantly reduced RV/tibia ratio (2.04 ± 0.42 mg/mm versus 2.55 ± 0.36 mg/mm of vehicle treated animals, *P* < 0.05) (Figure 1(d)). Additionally, treatment of animals with SB204741 in a dose 5 mg/kg body weight did not decrease RVP_sys_ (57.9 ± 15.1 mmHg versus 63.0 ± 10.7 mmHg of vehicle treated animals) (Figure 1(c)) but significantly reduced RV/tibia ratio (0.48 ± 0.13 mg/mm versus 0.55 ± 0.08 mg/mm of vehicle treated animals) (Figure 1(d)).

**Figure 2:**
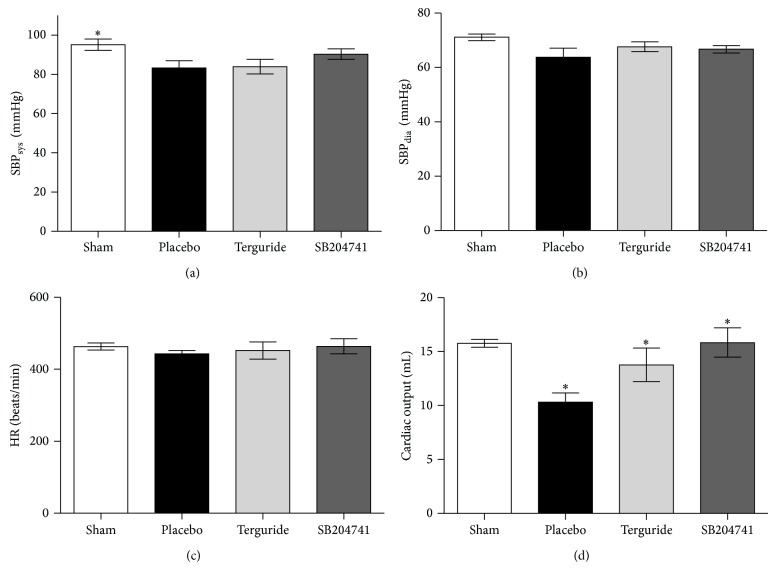
Effects of 5-HT2BR blockade on hemodynamics and cardiac performance. Hemodynamic catheter measurements of pulmonary artery banding (PAB) challenged mice showed no change in systolic (a) and diastolic (b) systemic blood pressure. Treatment with Terguride (0.2 mg/kg bodyweight) or SB204741 (5 mg/kg bodyweight) did not affect systemic parameters. Heart rate (HR) was not affected by PAB-operation or serotonin receptor blockade (c). Chronic constriction of the pulmonary artery decreased cardiac output compared to sham-operated controls significantly (d). Magnetic resonance imaging revealed significant cardiac performance improvement due to the administration of Terguride or SB204741 compared to placebo-treated animals. Data are presented as mean ± SEM. *n* = 16–18 animals in catheterized untreated control groups and *n* = 6–9 animals in all treated and MRI groups, respectively. ^*^ *P* < 0.05 versus placebo-treated control.

Systolic (SBP_sys_) and diastolic (SBP_dia_) systemic blood pressure were not different among the groups at day 21 after pulmonary artery constriction (Figures 2(a) and 2(b)). Pulmonary artery banding induced cardiac insufficiency as seen in cardiac output (10.3 ± 2.5 mL at day 21 in placebo-treated animals versus 15.8 ± 1.1 mL in sham-controls, *P* < 0.05) (Figure 2(d)) but did not affect heart rate (HR) in any of the above-mentioned groups (Figure 2(c)). Administration of Terguride improved cardiac function (13.8 ± 3.8 mL versus 10.3 ± 2.5 mL of vehicle treated animals, *P* < 0.05) (Figure 2(d)). Additionally, SB204741 attenuated PAB-induced insufficiency (15.9 ± 3.3 mL versus 10.3 ± 2.5 mL of vehicle treated animals, *P* < 0.05) (Figure 2(d)).

### 3.3. 5-HT2BR Antagonists Decrease RV Fibrosis after PAB

With respect to the cardiac collagen production, we found in RVs from PAB-operated mice 21 days after surgery a significant increase in the total collagen content (8.34 ± 1.36% in PAB-operated animals versus 0.80 ± 0.13% in sham-operated animals, *P* < 0.05) (Figures 1(e) and 1(f)). The collagen production in animals treated with Terguride reached values similar to those of sham-operated mice (3.90 ± 1.27% for Terguride treated animals versus 0.80 ± 0.13% in sham-operated and 8.34 ± 1.36% in vehicle treated animals, resp., *P* < 0.05) (Figures 1(e) and 1(f)). Similarly, the percentage of collagen amount was significantly reduced in SB204741-treated animals (2.90 ± 2.40% in SB204741-treated animals versus 8.34 ± 1.36% in vehicle treated animals, *P* < 0.05) (Figures 1(e) and 1(f)).

### 3.4. 5-HT2BR Antagonists Regulate Collagen Synthesis in Mouse Cardiac Fibroblasts

**Figure 3:**
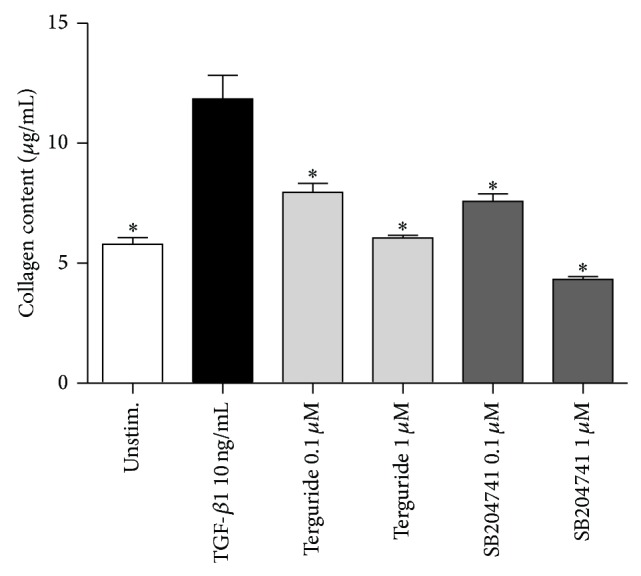
Effect of 5-HT2BR blockade on isolated murine cardiac fibroblasts. TGF-beta 1 induced production of collagen depends on the 5HT2BR. Cardiac fibroblasts were serum-starved and treated with Terguride or SB204741 at the concentrations indicated prior to TGF-beta 1 stimulations. Both 5-HT2BR antagonists significantly diminished the production of total secreted collagen of mouse RV cardiac fibroblasts to the medium. Data are presented as mean ± SEM, *n* = 4, ^*^ *P* < 0.05 versus TGF-beta 1 treated control.

To further strengthen the assumption that 5-HT2BR antagonists regulate collagen deposition, adult mouse cardiac fibroblasts were treated with Terguride or SB204741 followed by the stimulation with TGF-beta 1. Treatment with Terguride or SB204741 significantly reduced collagen secretion in comparison to TGF-beta 1 stimulation alone (11.84 ± 0.99 for TGF-beta 1 versus 6.06 ± 0.1 and 4.34 ± 0.11 for Terguride and SB204741, resp.). Our data demonstrate that both antagonists markedly decreased TGF-beta 1 induced total secreted collagen production of mouse cardiac fibroblasts* in vitro* (Figure 3).

## 4. Discussion

In this study we demonstrate that the 5-HT2A and 2B receptor antagonist Terguride and selective 5-HT2B receptor antagonist SB204741 attenuate RV remodeling in the surgical banding model of the pulmonary artery of right ventricular failure (RVF). Progressive RV remodeling, including increased wall thickness and myocardial hypertrophy as well as interstitial fibrosis, represents the main characteristics of pulmonary arterial hypertension (PAH) besides the pulmonary vascular remodeling and vasoconstriction processes in the lungs of affected patients [18]. Approved treatment strategies for PAH are mainly based on pulmonary vasodilation by employing prostanoids, endothelin receptor antagonists, phosphodiesterase (PDE) 5 inhibitors, and soluble guanylate cyclase (sGC) stimulators [19, 20]. Even while these therapeutic principles have convincingly proven efficacy in reducing the elevated pulmonary pressure and in improving the clinical function parameters such as the 6-minute walk test [21], their effects on the myocardial remodeling processes in pulmonary hypertension are only minor; the reduction in afterload is only a secondary effect of the vasodilation in the lung vasculature [22].

Clinical observations have provided evidence for the presence of increased systemic 5-HT-concentrations in idiopathic pulmonary arterial hypertension (IPAH) patients [23–26]. In the lung, effects of 5-HT are mainly mediated through the 5-HT transporter (5-HTT) and the 5-HT-receptor (5-HTR) isoforms [27]. It was shown that 5-HT2AR and 5-HT2BR are involved in the pathogenesis of PAH [28]. In the monocrotaline animal model for PAH beneficial effects of specific 5-HTR blockade was demonstrated [29–32]. Additionally, we have recently shown the effects of Terguride in monocrotaline injected rats with severe pulmonary hypertension [27]. It reduced pulmonary vascular remodeling and decreased therefore afterload and right heart hypertrophy. However, it is not known whether direct effects on the right ventricle contribute to these effects.

The pulmonary artery banding (PAB) procedure effectively induced RV hypertrophy, dysfunction, and failure [33]. These pathophysiological changes were characterized by myocardial structural changes, such as an increased collagen production of cardiac fibroblasts, that is, increased fibrosis. The primary aim of this study was to evaluate the histological and functional effects of serotonin 2B receptor antagonists in the pressure-overloaded right heart. The biochemical and cellular responses to cardiac hypertrophy and failure are yet to be fully explored [3]. Elucidating the functional and histological effects of pharmaceutical interventions on the development of cardiac hypertrophy and failure can help us to understand the underlying cellular mechanisms. Previous reports suggested that an interplay of TGF*β*3 and 5-HT signaling might exist, as the blockade of the 5HT2b receptor resulted in complete cessation of TGF*β*3 induced mechanical strengthening, suggesting TGF*β*/5-HT signaling as a potent mechanism for control of biomechanical remodeling of atrioventricular cushions during development [34]. Thus it is tempting to speculate that the effect of serotonin on 5-HT signaling is mediated by TGF-beta. Thus, the antagonism of the 5-HT2BR might even be more promising as a strategy for therapeutic intervention in RVF, since the 5-HT2B signaling is not restricted to the heart but has also been implicated in the development of pulmonary arterial hypertension (PAH) [14]. Inhibition of excessive receptor activation in the lung and in the heart in PAH provides a strong rationale for clinical evaluation of such agents in the treatment of PAH and RVF.

A key role for 5-HT2BR activation in left ventricular remodeling processes and the development and progression of heart failure has been suggested by [9, 10]. The 5-HT2BR expression in cardiac fibroblasts and their contribution to development of cardiac hypertrophy were shown in 2009 [5, 11]. Interestingly, Lairez et al. demonstrated in 2013 that, after transverse aortic constriction, left ventricular 5-HT2AR expression was transiently increased and selective blockade of the receptor prevented the development of cardiac hypertrophy [6].

Terguride is known as a high affinity modulator of neurotransmitter receptors including serotonin, dopamine, and *α*2-adrenergic receptors [35–37]. Here we have studied it as a prototypic drug for the therapeutic intervention by 5-HT2R blockade in RVF. This study demonstrates that Terguride and SB204741 (a) improve hemodynamics, (b) restore cardiac output, and (c) prevent and reverse cardiac fibrosis induced by PAB-operation in mice. Given our findings, blocking of the 5-HT2B receptor by specific antagonists may be a useful therapy for patients suffering from right heart disease and warrants for future clinical investigations.

## 5. Conclusion

In this study, we investigated whether a therapeutic intervention using 5-HT2B receptor antagonists Terguride and SB204741 exerts antifibrotic potency in chronic experimental pressure overload-induced RVF in mice. As serotonin receptor antagonism in experimental RVF has not been addressed by other research groups yet, we demonstrate that administration of both Terguride and SB204741 weakens myocardial structural changes by reducing RV hypertrophy and fibrosis in PAB mice. In conclusion, serotonin 2B receptor antagonism either by SB204741 or by Terguride decreased RV hypertrophy and improved RV contractile function when given to animals with established RV failure. Our data proposes that 5-HT2BR antagonists and, more specifically, Terguride, which is clinically approved and well tolerated, might serve as a new cardioprotective tool for the treatment of RVF.
